# Genetic Algorithm-Based Test Data Generation for Multiple Paths via Individual Sharing

**DOI:** 10.1155/2014/591294

**Published:** 2014-10-16

**Authors:** Xiangjuan Yao, Dunwei Gong

**Affiliations:** ^1^College of Science, China University of Mining and Technology, Xuzhou, Jiangsu 221116, China; ^2^School of Information and Electrical Engineering, China University of Mining and Technology, Xuzhou, Jiangsu 221116, China

## Abstract

The application of genetic algorithms in automatically generating test data has aroused broad concerns and obtained delightful achievements in recent years. However, the efficiency of genetic algorithm-based test data generation for path testing needs to be further improved. In this paper, we establish a mathematical model of generating test data for multiple paths coverage. Then, a multipopulation genetic algorithm with individual sharing is presented to solve the established model. We not only analyzed the performance of the proposed method theoretically, but also applied it to various programs under test. The experimental results show that the proposed method can improve the efficiency of generating test data for many paths' coverage significantly.

## 1. Introduction

One of the approaches to improve the quality of software is to do a large number of tests before delivery and usage in order to detect bugs or faults in software. Software testing is an expensive, tedious, and labor-intensive task and requires significant human effort [1]. If the process of testing can be automated, it will undoubtedly shorten the period of software development and improve the quality of software, so as to enhance the market competitiveness. One of the most important issues in automated software testing is the generation of effective test data satisfying the selected test adequacy criteria.

It has been proved that many software test problems can come down to those of generating test data for paths coverage [2, 3], which can be described as follows: for a given path of a program under test, search for a test datum in the input domain of the program, such that the traversed path of the test datum is just the desired one.

In recent years, it is becoming a promising direction to generate test data for complex software using the genetic algorithm (for short, GA) and has achieved many research results [4]. But most GA-based test data generation methods for path coverage intend to cover target paths one by one, which make the process of test data generation inefficient.

In this study, we established a mathematical model of generating test data for multiple paths coverage, which takes each optimization problem corresponding to one target path as a subproblem, and a number of subproblems form an overall optimization problem. This model is different from those existing multiobjective problems due to the specificity of generating test data.

On this basis, we proposed a multipopulation genetic algorithm to solve the proposed optimization problem. In our algorithm, each subpopulation optimizes one subproblem, so the fitness functions of different subpopulations differ from each other. All subpopulations evolve in parallel. A very key step of our algorithm is the individual sharing of different subpopulations; specifically, every time when the evolutionary operations of a generation finish, the algorithm not only determines whether an individual is an optimal solution of the subpopulation it belongs to, but also does that for the other subpopulations. By this way, the efficiency of finding optimal solutions for each subproblem improves with the complexity of the algorithm not increasing obviously.

We not only analyzed the performance of the proposed method theoretically, but also applied it to different programs under test for evaluation. The experimental results show that the proposed method can significantly improve the efficiency of generating test data for many paths' coverage.

This paper is divided into nine sections, and the remainder is organized as follows: Section 2 briefly reviews the related works; Section 3 gives a model of generating test data for multiple path coverage; a multipopulation genetic algorithm is proposed to solve the model in Section 4; Section 5 analyzes the performance of the proposed algorithm theoretically; the experiments are presented in Section 6; Section 7 discusses possible threats to the validity of the proposed method; finally, conclusion is presented in Section 8.

## 2. Related Work 

This section provides a survey on GA-based software testing. First, some basic methods of automatic software testing are introduced. Then, we review the main works on GA-based test data generation. Finally, we talk about the challenges of path coverage testing.

### 2.1. Automatic Software Testing

Since the process of software testing is highly time and resource consuming, many automatic approaches have been developed to facilitate the process and decrease its cost, which can be divided into four categories, namely, random method, static method, dynamic method, and heuristics method.

Random method generates test data by randomly sampling the input space of a program under test [5]. This approach is simple but has certain blindness in generating test data. Some improved methods have been proposed to heighten the diversity of test data [6, 7].

Static method only needs static analysis and transformation, without involving actual execution of the program under test, such as symbolic execution [2, 8], and domain reduction [9]. But this method usually requires a significant amount of algebra and (or) interval arithmetic [10].

Dynamic structural method of generating test data was firstly proposed by Miller and Spooner [11], which needs real execution of the program under test, in order to obtain useful information [3].

Different from dynamic method, the process of generating test data by heuristics method is not completely determined. Heuristics method usually recurs to some sort of heuristic algorithms, such as the genetic algorithm, simulated annealing, tabu search, and scatter algorithm, of which the GA is the most widely used [12].

### 2.2. GA-Based Test Data Generation

As an efficient search-based optimization algorithm, the GA shows special advantage and efficiency in solving problems with high complexity, such as the problems of large space, multipeak, and nonlinear. Therefore it has become a research hotspot to automatically generate test data with GAs and produced encouraging results [13].

Gong and Yao [14] used a GA to generate test data for statement coverage based on testability transformation. Yao et al. [15] proposed an approach to reduce target statements according to their dominant relations and the test suite covering the reduced set of target statements was generated by a GA.

Miller et al. [16] used GAs to generate test data satisfying branch coverage criterion. The experimental results show that the test suite obtained by GAs can achieve or be very close to branch coverage. Baars et al. [17] presented an algorithm for constructing fitness functions that improve the efficiency of search-based testing when trying to generate branch adequate test data. Alshraideh et al. [18] proposed a multiple-population algorithm to improve the efficiency of branch coverage testing. The experimental results showed that the proposed method outperforms the single-population algorithm significantly.

Michael et al. [19] used a GA to generate test data satisfying condition coverage criterion. In their work, the problem of test data generation is reduced to a function minimization, and the function is minimized using one of two genetic algorithms in place of the local minimization techniques.

As for the works of GA-based software testing for path coverage criterion, we will introduce them individually in Section 2.3.

Besides traditional structural software testing, Bühler and Wegener [20] applied an evolutionary algorithm to functional testing. Watkins and Hufnagel [21] used two GAs to generate a couple of test data pieces and then trained a decision tree using them, in order to obtain an agent model which distinguishes the merit of test data. Ferrer et al. [22] presented a method of automatically generating test data by considering multiple objectives: maximizing the coverage and minimizing the oracle cost.

### 2.3. GA-Based Path Testing

Path coverage testing is the strongest sufficiency criterion in white box testing. Automatically generating data for paths coverage remains a challenging problem [23].

Bueno and Jino [24] and Watkins and Hufnagel [25] used a GA to obtain test data fulfilling path coverage, respectively. Mei and Wang [26] proposed a method that can automatically generate test cases for selected paths using a special genetic algorithm. In their algorithm, the best chromosome called queen crosses with the selected drones, which enhances the exploitation of global optimal solutions.

Hermadi and Ahmed [27] have observed that existing GA-based test data generators can generate only one test datum for one test goal at a time. When there are many target paths to be covered, the generator has to be run many times. In fact, the generated individuals when trying to find test data covering a path may be just test data covering other target paths. This, hence, makes those existing test data generators inefficient in trying to generate test data for multiple paths.

Wegener et al. [28] developed a fully automatic GA-based test data generator for structural software testing. In their approach, all generated individuals are evaluated with regard to all unachieved partial aims. Partial aims reached by chance are identified, and the individuals with good fitness values for one or more partial aims are noted and stored for seeding the subsequent testing of uncovered targets. But they only considered one partial aim for optimization at a time, which means that they solved the problems of generating test data one by one. Furthermore, they did not discuss whether multiple targets can be covered in one run. Besides, they reported that full coverage of some programs is achieved but not for all programs though.

Bueno and Jino [24] looked after methods to improve the performance of test data generation by using past input data to compose the initial population for the search. Although these methods can improve the performance of the initial population by reusing test data, they still cannot make full use of the test data generated in the evolutionary process.

Ahmed and Hermadi [29] proposed a GA-based test data generator for multiple paths. In their work, the problem of generating test data for multiple paths is regarded as a multiobjective optimization problem and solved by a multiobjective evolutionary algorithm. In fact, the problem of generating test data for multiple paths is strictly different from traditional multiobjective optimization problems. Therefore, it is necessary to establish an appropriate mathematical model for the problem of generating test data for multiple paths coverage according to its specificity and give a corresponding evolutionary solution.

Gong and Zhang [30] also proposed a test data generation method for multipath coverage. They represent a target path using Huffman encoding method and designed the fitness function according to the Huffman codes of target paths. Their method is simple and has better performance than Ahmed's method, but the fitness function cannot distinguish individuals well.

In order to stop searching as soon as all feasible paths have been covered, Hermadi et al. [31] proposed method for determining when it is no longer worthwhile to continue searching for test data to cover uncovered target paths. Compared to searching for a standard number of generations, an average of 30–75% of total computation was avoided in test programs with infeasible paths, and no feasible paths were missed due to early termination. The extra computation in programs with no infeasible paths was negligible.

## 3. Mathematical Model of Test Data Generation for Multiple Paths 

In order to illustrate conveniently, we first introduce several concepts. Then, an objective function is constructed in order to transform the problem of generating test data into an optimization one. On this basis, the optimization model of generating test data for multiple paths coverage is established.

### 3.1. Basic Concepts

*Control Flow Graph (CFG) [1]*. The CFG of a program Φ is a directed graph *G* = (*N*, *E*, *s*, *e*), where *N* is the set of nodes, *E* is the set of edges, and *s* and *e* are unique entry and exit nodes of the graph, respectively. Each node *n* is a statement in the program; each edge (*n*_*i*_, *n*_*j*_) represents a transfer of control from node *n*_*i*_ to node *n*_*j*_.

*Path [1]*. A path of a CFG is a sequence *P* = *n*_1_, *n*_2_,…, *n*_*k*_, such that there exists an edge from node *n*_*i*_ to *n*_*i*+1_, *i* = 1,2,…, *k* − 1.

For large-scale programs, the sequence of a path may be very long. We represent a path using a (0, 1)-string for simplicity. Suppose that there are *m* conditional statements in path *P*, denoted as *C*_1_, *C*_2_,…, *C*_*m*_. Define

(1) $$\begin{array}{c} \gamma_{i}=\{\begin{array}{cc} 0, & P\;\;\text{inludes}\;\text{the}\;\text{false}\;\text{branch}\;\text{of}\;\;C_{i}\\ 1, & P\;\;\text{inludes}\;\text{the}\;\text{true}\;\text{branch}\;\text{of}\;\;C_{i}.\; \end{array} \end{array}$$

Thus we obtain a (0-1)-string *γ*_1_ *γ*_2_ ⋯ *γ*_*m*_ of length *m*. In program Φ, the mapping between a path and such a (0, 1)-string is one to one. Without special illustration, a path is represented by such a (0, 1)-string in this study.

Let the input vector of program Φ be *X* = (*x*_1_, *x*_2_,…, *x*_*s*_), and let the domain of *x*_*i*_ be *D*_*i*_; then the* input domain of *Φ is *D*(Φ) = *D*_1_ × *D*_2_ × ⋯×*D*_*s*_. When program Φ adopts *X* as an input, the traversed path is denoted by *P*(*X*). We call the first dissimilar character of *P* and *P*(*X*) their* bifurcation*.

### 3.2. Structure of Objective Function

The key problem of applying GAs to test data generation is the construction of a suitable objective function. The goodness of a candidate test datum is often expressed in terms of the closeness that the test datum fulfills the test goal. The approach to forming an objective function typically involves two parts:* approach level* (*AL*) and* branch distance* (*BD*) [3, 24, 25].

The approach level assesses how close an execution comes to reaching the predicate which controls the test object. If *P* ≠ *P*(*X*), we define the approach level of input *X* to a target path *P* as the number of characters between the bifurcation of *P* and *P*(*X*) to the last character of *P*, denoted by *AL*_*P*_(*X*); otherwise, we define *AL*_*P*_(*X*) = 0. *X* covers path *P* if and only if *AL*_*P*_(*X*) = 0.

For example, suppose that *P* = 1001001 is a target path, *P*(*X*_1_) = 1001110, and *P*(*X*_2_) = 1001001; then *AL*_*P*_(*X*_1_) = 3 and *AL*_*P*_(*X*_2_) = 0.

The branch distance assesses how close the predicate comes to evaluating either true or false branch. For example, suppose that a conditional statement is “if *a* ≥ 12,” and the aim is to execute the true branch. Suppose that the value of *a* is *a*(*X*) after the execution of this statement with input *X*; then the branch distance of *X* for branch condition *a* ≥ 12 is defined as follows:

(2) $$\begin{array}{c} BD\left(X,a\geq 12\right)=\{\begin{array}{cc} 0 & \text{if}\;\;a\left(X\right)\geq 12,\\ 12-a\left(X\right) & \text{others}. \end{array} \end{array}$$

Branch distances of different kinds of simple branch conditions are listed in Table 1. For a complex branch condition, branch distance is the composite of those of all simple conditions included in it, which is listed in Table 2.

We define the general objective function *f*_*P*_(*X*) of input *X* to target path *P* as follows:

(3) $$\begin{array}{c} f_{P}\left(X\right)=AL_{P}\left(X\right)+\text{normalized}\left(BD_{P}\left(X\right)\right), \end{array}$$

where *BD*_*P*_(*X*) refers to the branch distance of *X* to the conditional statement corresponding to the bifurcation of *P* and *P*(*X*), and function

(4) $$\begin{array}{c} \text{normalized}\left(x\right)=1-1.01^{-x}. \end{array}$$

Function normalized maps the value of *BD*_*P*_(*X*) to interval [0,1).

A sufficient and necessary condition of *f*_*P*_(*X*) = 0 is that the traversed path of *X* is *P*; that is, *P*(*X*) = *P*; furthermore, the smaller the value of *f*_*P*_(*X*), the nearer the *X* to the data covering *P*. So the problem of generating test data for path *P* can be transformed into that of minimizing *f*_*P*_(*X*).

**Figure 1:**
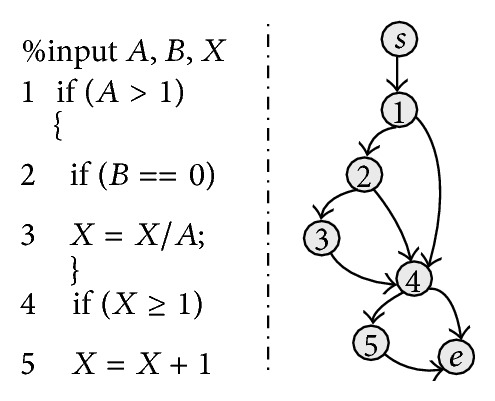
An example program and its CFG.

For example, see the program in Figure 1. Suppose that the target path is $P=\begin{array}{ccccccc} s & 1 & 2 & 3 & 4 & 5 & e \end{array}$. There are three conditional statements in *P*, that is, statements 1, 2, and 4, respectively. *P* traverses the true branches of all these statements. So we also write *P* = 111. Suppose that *X*_1_ = (−10,5, 10), *X*_2_ = (2,5, 10), and *X*_3_ = (2,1, 10). We obtain *P*(*X*_1_) = 01, *P*(*X*_2_) = 101, and *P*(*X*_3_) = 101. Thus *AL*_*P*_(*X*_1_) = 3 − 0 = 3, *AL*_*P*_(*X*_2_) = 3 − 1 = 2, and *AL*_*P*_(*X*_3_) = 3 − 1 = 2.

In addition, *P*(*X*_1_) deviates *P* from the first conditional statement, so the branch distance of *X*_1_ to *P* is *BD*_*P*_(*X*_1_) = 11; similarly, we get *BD*_*P*_(*X*_2_) = 5 and *BD*_*P*_(*X*_3_) = 1. Thus

(5) $$\begin{array}{c} f_{P}\left(X_{1}\right)=3+1-1.01^{-11}=3.1037,\\ f_{P}\left(X_{2}\right)=2+1-1.01^{-5}=2.0485,\\ f_{P}\left(X_{3}\right)=2+1-1.01^{-1}=2.0099. \end{array}$$

Although the traversed paths of *X*_2_ and *X*_3_ are the same, the branch distance of *X*_3_ is smaller than that of *X*_3_. Thus *X*_3_ obtains a better objective value than *X*_2_.

### 3.3. Mathematical Model of Generating Test Data for Multiple Paths Coverage

Let the set of target paths be Γ = {*P*_1_, *P*_2_,…, *P*_*n*_}; then the problem of generating test data for Γ can be described as follows: find a test suite {*X*_1_, *X*_2_,…, *X*_*n*_}, such that *P*(*X*_*i*_) = *P*_*i*_. Let the objective function for path *P*_*i*_ using the method proposed in Section 3.2 be *f*_*i*_(*X*); then the problem of generating test data for {*P*_1_, *P*_2_,…, *P*_*n*_} can be transformed into an optimization one described as follows:

(6) min⁡ f1(X)s.t.  X∈D(Φ)min⁡ f2(X)s.t.  X∈D(Φ)   ⋮min⁡ fn(X)s.t.  X∈D(Φ).

Most existing GA-based test data generation methods take the above problem as *n* self-governed optimization ones and solve them one by one. Specifically, for each optimization problem min⁡*f*_*i*_(*X*), run a GA in order to find an optimal solution of *f*_*i*_(*X*), which is just a test datum traversing target path *P*_*i*_. Repeat above process, until all optimization problems have been solved. If the number of target paths is *n*, the GA has to be run *n* times.

This approach, however, does not take advantage of the fact that some of the required test data can be readily available as by-products when trying to find other test data, because different target paths have similarities. Therefore the efficiency of these methods is low when *n* is large.

Ahmed et al. gave an algorithm of generating test data for multiple paths coverage, but they regarded this problem as a multiobjective optimization one. Thus, their model should be

(7) min⁡ F(X)=(f1(X),f2(X),…,fn(X))s.t. X∈D(Φ).

In fact, the problem of generating test data for multiple paths is strictly different from traditional multiobjective optimization ones. In traditional multiobjective optimization problems, the aim is to find one solution which satisfies all objectives well. In a multiobjective environment, we often encounter conflicting objectives with some trade-off among them. But for the problem of generating test data for paths *P*_1_, *P*_2_,…, *P*_*n*_, what we need is to obtain a test suite {*X*_1_,…, *X*_*n*_}, where *X*_*i*_ is an optimal solution of *f*_*i*_(*X*), *i* = 1,…, *n*.

In addition, the number of objective functions in traditional multiobjective optimization problems remains unchanged, while that in the proposed model gradually reduces. Therefore, there is much limitation to take the problem of generating test data as a multiobjective optimization one.

Different from existing methods, we consider the problem of generating test data for *n* paths coverage as a uniform problem, in which each optimization problem corresponding to one target path is a subproblem. We solve all subproblems at the same time. Thus the problem corresponding to the test data generation for multiple paths coverage can be described as follows:

(8) min⁡ f1(X1)min⁡ f2(X2)  ⋯min⁡ fn(Xn)s.t.  X1,X2,…,Xn∈D(Φ).

This model includes *n* subproblems, each of which is a minimization problem, and all objective functions have the same domain. We will seek an algorithm to solve these *n* problems simultaneously, rather than solve them independently. So problem (8) strictly differs from (6) and (7), and we should seek a suitable method to solve it.

## 4. Multipopulation GA for Test Data Generation of Multiple Paths 

In this section we will give a multipopulation GA to solve problem (8), which is different from traditional multipopulation GAs. The main purpose of our strategy is to expand the search range of each population by individual sharing, so as to improve the efficiency of the algorithm.

### 4.1. Initialization of Populations

For the *i*th optimization problem min⁡*f*_*i*_(*X*_*i*_), randomly generating a subpopulation of size *m*, that is, *ℵ* ^(1)^(*P*_*i*_) = {*X*_*i*1_^(1)^, *X*_*i*2_^(1)^,…, *X*_*im*_^(1)^}, *i* = 1,…, *n*, where *X*_*ij*_^(1)^ refers to the *j*th individual in the *i*th population of the first generation. An individual corresponds to a string by proper encoding. Population size might have some influence on the performance of the algorithms, but this is not a focus of this study, so we just give an appropriate value for it.

### 4.2. Genetic Operations

As a typical GA, our method mainly includes three kinds of operations, that is, selection, crossover, and mutation.

Individuals are selected according to their fitness, so that good gens have more chances to be copied to the next generation. We adopt objective function *f*_*i*_(*X*_*ij*_^(1)^) as the fitness of individual *X*_*ij*_^(1)^. Because what we are solving are minimization problems, the smaller the fitness of an individual is, the better we consider it.

Crossover operation exchanges parts of two gene strings in a certain probability to produce two new chromosomes, while mutation operation modifies some of the genes in a string, resulting in a new chromosome. The crossover and mutation rates are denoted by *P*_*c*_ and *P*_*m*_, respectively. Because parameter setting is not the focus of this work, we just give the value of the parameters based on experience.

**Figure 2:**
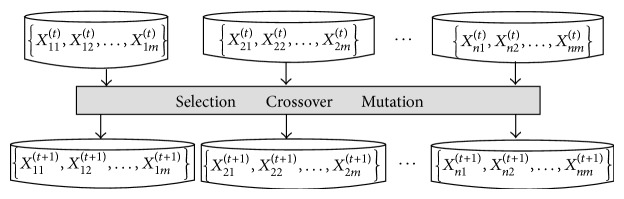
Evolution of individuals in our multipopulation genetic algorithm.

Each subpopulation implements these operations independently. By this way, individuals of the *t*th generation are evolved to the (*i* + 1)th generation, which can be shown as Figure 2.

### 4.3. Individual Sharing among Different Subpopulations

**Figure 3:**
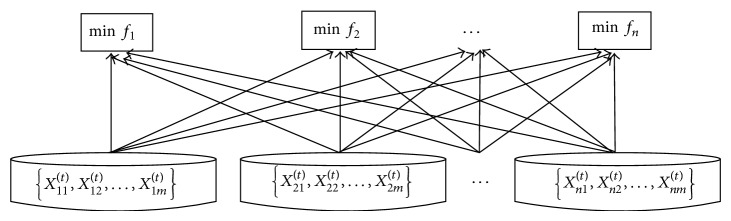
Individual sharing among subpopulations.

The biggest difference between traditional multipopulation GAs and the proposed one lies in the following: in traditional multipopulation GAs, subpopulations communicate by means of individual migration, while in our method, subpopulations communicate through individual sharing among subpopulations. Specifically, every time when the evolutionary operations of a generation finish, the algorithm not only determines whether an individual is an optimal solution of the subpopulation it belongs to, but also does that for the other subpopulations. In this way, the individuals of one subpopulation are shared by all other subpopulations, and the probability of finding optimal solutions significantly increases. The implementation of individual sharing is shown as Figure 3.

Because *AL*_*p*_(*X*) = 0 if and only if the traversed path of *X* is just *P*, we determine whether *X* is a desired test datum covering *P* according to the value of *AL*_*P*_(*X*). Suppose that there are *n* target paths *P*_1_,…, *P*_*n*_. In our algorithm, we can obtain the values of *AL*_*P*_1__(*X*),…, *AL*_*P*_*n*__(*X*) in one run of the instrumented program with input *X*. Thus the individual sharing can be realized with the computation complexity not increasing too much.

### 4.4. Steps of the Algorithm

Based on the above discussion, the main steps of the proposed algorithm are shown as follows.

**Step 1 .**

Set the values of the number of subpopulations *n*, maximum termination generation *T*, crossover probability *P*_*c*_, and mutation probability *P*_*m*_, where *n* is equal to the number of target paths.

**Step 2 .**

Suppose that the set of target paths is {*P*_1_, *P*_2_,…, *P*_*n*_}. For *P*_*i*_, randomly generate a subpopulation *ℵ* ^(1)^(*P*_*i*_) = {*X*_*i*1_^(1)^, *X*_*i*2_^(1)^,…, *X*_*im*_^(1)^}, *i* = 1,2,…, *n*. The value of generation *t* = 1.

**Step 3 .**

For subpopulation *ℵ* ^(*t*)^(*P*_*i*_) in the *t*th generation, calculate the values of

(9) $$\begin{array}{c} AL_{P_{1}}\left(X_{ij}^{\left(t\right)}\right),\ldots ,AL_{P_{n}}\left(X_{ij}^{\left(t\right)}\right) \end{array}$$

for individual *X*_*ij*_^(*t*)^ to all target paths and those of *BD*_*P*_*i*__(*X*_*ij*_^(*t*)^) for individual *X*_*ij*_^(*t*)^ to path *P*_*i*_, *i* = 1,2,…, *n*, *j* = 1,2,…, *m*.

**Step 4 .**

*f*_*i*_(*X*_*ij*_^(*t*)^) = *AL*_*P*_*i*__(*X*_*ij*_^(*t*)^) + normalized(*BD*_*P*_*i*__(*X*_*ij*_^(*t*)^)) is used as the fitness of individual *X*_*ij*_^(*t*)^ for subpopulation *ℵ* ^(*t*)^(*P*_*i*_) to guide the evolution.

**Step 5 .**

If there is a *AL*_*P*_*k*__(*X*_*ij*_^(*t*)^) = 0, which means that *X*_*ij*_^(*t*)^ covers *P*_*k*_, then *X*_*ij*_^(*t*)^ is an optimal solution of the *k*th optimization subproblem. In this case, save *X*_*ij*_^(*t*)^, delete *P*_*k*_ from the target path set, and terminate the evolution of the *k*th subpopulation.

**Step 6 .**

If the number of subpopulations becomes 0, or the number of generations is larger than *T*, then stop the evolution and output the test data; otherwise, go to Step 7.

**Step 7 .**

Perform genetic operations on *ℵ* ^(*t*)^(*P*_*i*_) to generate offspring population *ℵ* ^(*t*+1)^(*P*_*i*_). let *t* = *t* + 1 and go to Step 3.

## 5. Performance Analysis 

We will illustrate the performance of the proposed algorithm by analyzing its efficiency and computational complexity.

### 5.1. Efficiency of Algorithm

Suppose that the set of target paths is {*P*_1_, *P*_2_,…, *P*_*n*_}  (*n* > 1) and *ℵ*(*P*_*i*_) is the subpopulation used to optimize the *i*th subproblem, which is related to the problem of generating test data for *P*_*i*_. Let *T*_*i*_ be the number of generations in which the *i*th subpopulation finds the test datum covering path *P*_*i*_; thus *T*_*i*_ is a random variable. From experiences, we can suppose that *T*_*i*_ ~ *N*(*μ*_*i*_, *σ*_*i*_^2^). Let *T*_*ij*_, *i* ≠ *j*, be the number of generations in which the *i*th subpopulation finds the test datum covering *P*_*j*_; then *T*_*ij*_ is also a random variable. Suppose that the probability of *ℵ*(*P*_*i*_) finding the test datum that covers *P*_*j*_ is *λ*_*j*_; then *P*{*T*_*ij*_ = *t*} = (1 − *λ*_*j*_)^*t*−1^ *λ*_*j*_, *t* = 1,2,…. For convenience to illustration, we also denote *T*_*i*_ by *T*_*ii*_.

If we use traditional single-objective GAs to solve (3), in the circumstance of using the same population size, the probability of *ℵ*(*P*_*i*_) finding an optimal solution within *t* generations is *P*{*T*_*i*_ ≤ *t*} = Φ((*t* − *μ*_*i*_)/*σ*_*i*_), where Φ(*x*) is the distribution function of standard normal distribution. Thus the probability of all subpopulations finding their optimal solutions within *t* generations is

(10) Γ1(t)=P{T1≤t,…,Tn≤t}=P{T1≤t}⋯P{Tn≤t}=∏i=1nΦ(t−μiσi).

If we adopt the proposed method to solve (6), then the probability of finding the test datum covering path *P*_*i*_ within *t* generations is

(11) P{min⁡(T1i,…,Tni)≤t}  =1−P{min⁡(T1i,…,Tni)>t}  =1−P{T1i>t}⋯P{Tni>t}  =1−(1−P{T1i≤t})   ×⋯×(1−P{Tni≤t})  =1−[1−Φ(t−μiσi)](1−λi)t(n−1).

Thus the probability of all subpopulations finding all optimal solutions within *t* generations is

(12) $$\begin{array}{c} \Gamma_{2}\left(t\right)=\overset{n}{\underset{i=1}{\prod }}\left\{1-\left[1-\Phi \left(\frac{t-\mu_{i}}{\sigma_{i}}\right)\right]{\left(1-\lambda_{i}\right)}^{t(n-1)}\right\}. \end{array}$$

Since (1 − *λ*_*i*_)^*t*(*n*−1)^ < 1, we obtain

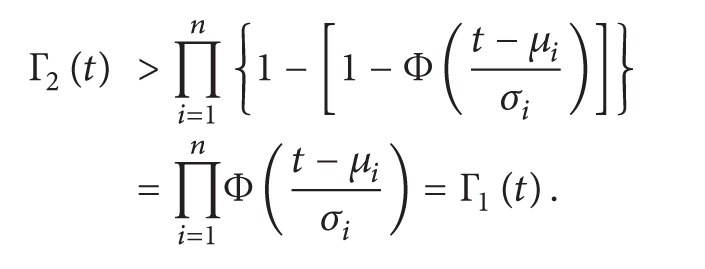
(13)


That is to say, the probability of finding all optimal solutions using the proposed algorithm is larger than that of traditional single-objective GAs. In addition, the more the number of target paths is, the more obvious the advantage of the proposed method is, which can also be easily understood by the following example.

Suppose that the set of target paths is {*P*_1_,…, *P*_5_} and *T*_*i*_ ~ *N*(500,100^2^), *λ*_*j*_ = 1/10000, *i*, *j* = 1,…, 5; then the probabilities of finding all optimal solutions within 500 and 600 generations using traditional single-objective GAs are

(14) Γ1(500)=∏i=15Φ(500−500100)=0.55=0.0313,Γ1(600)=∏i=15Φ(600−500100)=0.81435=0.3580,

respectively, whereas the probabilities of finding all optimal solutions within 500 and 600 generations using the proposed algorithm are

(15) Γ2(500)=∏j=15{×(1−110000)500×(5−1)1−[1−Φ(500−500100)]     × (1−110000)500×(5−1)}=0.59065=0.0719,Γ2(600)=∏j=15{×(1−110000)600×(5−1)1−[1−Φ(600−500100)]     × (1−110000)600×(5−1)}=0.85395=0.4540,

respectively. If the number of target paths increases to 10, and *T*_*i*_ ~ *N*(500,100^2^), *λ*_*j*_ = 1/10000, *i*, *j* = 1,…, 10, then the probabilities of finding all optimal solutions within 500 and 600 generations using traditional single-objective GAs are

(16) $$\begin{array}{c} \Gamma_{1}\left(500\right)=\overset{10}{\underset{i=1}{\prod }}\Phi \left(\frac{500-500}{100}\right)=0.5^{10}=0.000977,\\ \Gamma_{1}\left(600\right)=\overset{10}{\underset{i=1}{\prod }}\Phi \left(\frac{600-500}{100}\right)=0.8143^{10}=0.1282, \end{array}$$

respectively, whereas the probabilities of finding all optimal solutions within 500 and 600 generations using the proposed algorithm are

(17) Γ2(500)=∏j=110{(1−110000)500×(10−1)1−[1−Φ(500−500100)]     × (1−110000)500×(10−1)}=0.681210=0.0215,

(18) Γ2(600)=∏j=110{(1−110000)600×(10−1)1−[1−Φ(600−500100)]     × (1−110000)600×(10−1)}=0.891810=0.3182,

respectively. As can be seen from these results, in circumstance with 5 target paths, the probabilities of finding all optimal solutions within 500 and 600 generations using the proposed algorithm are 0.0719 and 0.4540, respectively, which are 0.0719/0.0313 ≈ 2.3 and 0.4540/0.3580 ≈ 1.3 times those of traditional single-objective GAs; in circumstance with 10 target paths, the probabilities of finding all optimal solutions within 500 and 600 generations using the proposed algorithm are 0.0215 and 0.3182, respectively, which are 0.0215/0.000977 ≈ 22 and 0.3182/0.1282 ≈ 2.5 times those of traditional single-objective GAs. The above results forcefully illuminate that the proposed algorithm is more efficient than traditional single-objective GAs; moreover, with the increase of the number of target paths, the advantages become more obvious.

### 5.2. Computational Complexity

We will compare the computational complexity of our multipopulation genetic algorithm and those of traditional ones. Suppose that the program under test has *l* executable statements and there are *n* target paths {*P*_1_, *P*_2_,…, *P*_*n*_}. The population size is *m*. Because *m* can be set manually, we consider *m* as a constant. We take the number of executed statements for the calculation of individual fitness and individual sharing in a generation as a measure of the computational complexity of an algorithm.

If we use traditional multipopulation GAs to solve the problem, which means that there is no individual sharing among subpopulations, then the program under test will be run *nm* times, which is equal to the number of all individuals. Since each run of the program probably executes *l* statements, the number of executed statements for the run of the program under test will be *lmn*. Taking the computation of the fitness as one statement, then all these *mn* individuals need to execute *mn* statements. So the number of executed statements in a generation using traditional multipopulation GAs is *C*(*l*, *n*) = *lmn* + *mn*.

If we use the proposed method to solve the problem, which means that subpopulations share all individuals, in addition to the run of the program under test and the computation of the fitness function, we consider the computation due to individual sharing among subpopulations. Taking the computation of the approach level as a statement, individual sharing needs to execute *mn* ^2^ sentences. So the number of executed sentences in a generation using the proposed method is *C*′(*l*, *n*) = *lmn* + *mn* + *mn* ^2^. Under normal circumstances, *n* is much smaller than *l*, so *lmn* + *mn* + *mn* ^2^ ≪ 2*lmn* + *mn*. Thus

(19) $$\begin{array}{c} \frac{C^{\prime }\left(l,n\right)}{C\left(l,n\right)}=\frac{lmn+mn+mn^{2}}{lmn+mn}\leq \frac{2lmn+mn}{lmn+mn}<2. \end{array}$$

On the other hand, each subpopulation has *m* individuals as possible solutions for each generation in traditional multipopulation GAs. But in our method, the possible solutions become *mn* for each generation via individual sharing, which is *n* times that of traditional methods. In other words, the population size is magnified to *n* times via individual sharing with the computation quantity almost doubling.

## 6. Experiments 

A group of experiments are conducted so as to investigate the performance of the proposed method. In the following section, subject programs are first introduced. Afterwards, experimental design is characterized. Finally, empirical results are presented and discussed.

### 6.1. Subject Programs

In order to evaluate the proposed method, we select eighteen programs for experiments.  Table 3 shows some basic information of each program, including its name, size, and description.  Table 3 is sorted by the sizes of the programs. These test subjects include not only laboratory programs, but also nontrivial industry ones. In addition, their lengths and functions are different from each other. These programs have been thoroughly used by other researches in the literature of software testing and analysis [19, 32–34]. The number of target paths for each program is also listed in Table 3.

For each program under test, we just randomly choose a part of feasible paths to cover. If there are too many paths to be covered, we can divide them into several groups, so that the scale of paths is reasonable. In addition, if we choose infeasible paths as target ones, the performances of different methods will not be distinguished, because it is impossible for any method to generate test data covering infeasible paths. The prediction of the infeasibility of a program path is an undecidable problem, and heuristic techniques that automatically select likely feasible paths can be employed [32].

### 6.2. Experimental Design

When designing the experiment, we specially have concern about two issues that can be described as follows.

**Proposition 1 .**

Can individual sharing improve the efficiency of the algorithm?

In order to verify the first proposition, we conduct two groups of experiments. In the first group of experiments, we use the proposed multipopulation GA with individual sharing to generate test data, while in the second one, different populations do not implement individual sharing but evolve independently.

**Proposition 2 .**

How is the overall performance of the proposed method?

In order to validate the overall performance of the proposed method in this study (for short, our method), we compare it with other three methods, namely, Gong's method [30], Ahmed's method [29], and random method. The reason why we adopt Gong's and Ahmed methods to compare is that they are also about the problem of generating test data for multiple paths. In addition, random method is a basic test technique and has been widely used, so we also adopt it as a consult object.

All methods (except random one) apply the same values of parameters, which are listed in Table 4. There are two termination criteria: one is that the test data for all target paths have been found; the other is that the number of generations has reached the maximum.

### 6.3. Experimental Results

In each group of experiments, we performed 30 runs for each program under test and record the time consumption of each run and each method, where the time consumption refers to the time needed to generate test data covering all target paths.

#### 6.3.1. Experimental Results for Testing the Performance of Individual Sharing

The experimental results to test the performance of individual sharing are listed in Table 5, in which Ave. and S.D. are the sample average and standard deviation of time consumption for each program and method, respectively. Sh.R. means the radio of the number of test data obtained by individual sharing and the number of all test data.

It can be seen from Table 5 that, (1) for all subject programs, the average time consumption using the method of individual sharing is all less than that not implementing individual sharing. The least time consumption of the method applying individual sharing is 6.38 seconds (Bubble Sort), while that not implementing individual sharing for the same program is 11.03 seconds. The most time consumption of the method applying individual sharing is 183.53 seconds (barcode), while that of the second method is 265.72 seconds. (2) The sharing rates of all programs exceed 30% except schedule (29.8%). The average sharing rate of the eight programs is 36.87%, which means that approximately one of each three test data pieces is obtained by individual sharing. By this way, we can make more full use of individuals generated in evolutionary process, therefore improving the efficiency of generating test data.

We use hypothesis testing to give a more scientific analysis for the above experimental results. Let *X*_1_ and *X*_2_ denote the time consumption using and not using individual sharing, respectively (without confusion, we will use the same symbol for all programs under test). It can be verified that *X*_1_ and *X*_2_ are random variables obeying normal distribution. Suppose that *X*_*i*_ ~ *N*(*μ*_*i*_, *σ*_*i*_^2^), *i* = 1,2. Because the sample standard deviation is an unbiased estimate of the standard deviation of the population, we take the values of sample standard deviations as those of standard deviations. Let the significance level *α* = 0.01. We will illustrate the performances of different methods by comparing *μ*_1_( = *E*(*X*_1_)) and *μ*_2_( = *E*(*X*_2_)).

**Step 1 .**

Establishing hypothesis:

(20) $$\begin{array}{c} H_{0}:\mu_{1}-\mu_{2}\geq 0;\;\;H_{1}:\mu_{1}-\mu_{2}<0. \end{array}$$

**Step 2 .**

Constructing statistics:

(21) $$\begin{array}{c} U=\frac{{\bar{X}}_{1}-{\bar{X}}_{2}}{\sqrt{\sigma_{1}^{2}/n_{1}+\sigma_{2}^{2}/n_{2}}}. \end{array}$$

**Step 3 .**

Giving rejection region:

(22) $$\begin{array}{c} U=\frac{{\bar{X}}_{1}-{\bar{X}}_{2}}{\sqrt{\sigma_{1}^{2}/n_{1}+\sigma_{2}^{2}/n_{2}}}\leq -Z_{\alpha }, \end{array}$$

where *α* = 0.01,  *n*_1_ = *n*_2_ = *n*_3_ = 30.

**Step 4 .**

Calculating the value of statistics.

The values of statistics *U* of different programs are listed in Table 6; *Z*_*α*_ = 2.325.

**Step 5 .**

Drawing conclusions

From Table 6 we conclude that the values of *U* are all less than −*Z*_*α*_ = −2.325. Then we reject null hypothesis *H*_0_ for all object programs, which means that the time consumption using individual sharing is significantly less than that not using it.

#### 6.3.2. Experimental Results for Testing the Proposed Method

The experimental results of comparing different methods are listed in Table 7. The meanings of all symbols are the same with Table 5. We also use hypothesis testing to give a scientific analysis for the above experimental results. The value of *U*_1_ shows the hypothesis testing results by comparing our method and Gong's method, that of *U*_2_ shows the hypothesis testing results by comparing our method and Ahmed's method, and that of *U*_3_ shows the hypothesis testing results by comparing our method and the random method.

It can be seen from Table 7 that, (1) for all subject programs, the average time consumption using our method is all less than that of Gong's, Ahmed's, and the random methods. The least time consumption of our method is 5.85 seconds (Bubble Sort), while that of Gong's, Ahmed's, and the random methods for the same program is 8.79, 9.85, and 12.32 seconds, respectively. The largest time consumption of our method is 192.82 seconds (Barcode), while that of Gong's, Ahmed's, and the random methods is 224.87, 289.67, and 316.42 seconds, respectively. (2) Gong's and Ahmed's methods have better results than the random method but are all poorer than ours. (3) The values of *U*_2_ and *U*_3_ are all less than −*Z*_*α*_ = −2.325. The values of *U*_1_ are all less than −*Z*_*α*_ = −2.325 except three programs, that is, Comn, Splinge, and Printtok. For these three programs, the time consumption of our method is still all less than that of Gong's method. Then we conclude that the time consumption using our method is significantly less than that using Gong's, Ahmed's, and random methods.

## 7. Threats to Validity 

The present study focuses on generating test data for multiple paths coverage. One possible threat to the validity of the proposed method may be related to parameter settings. The settings of parameters in GAs have an influence on the performance of generating test data. Appropriate choices of these values can improve the performance of an algorithm and therefore enhance its efficiency in generating test data. However, how to set proper parameters is not the emphasis of this study; thus we just give the values of the parameters based on our experience. The second threat to the validity may have relation with the use of software systems. Thus, possible bugs or errors, different program conversions, and test objectives may also have influence on the obtained results. Additionally, the selection of target paths may have also influenced the obtained results.

## 8. Conclusion 

We establish a mathematical model which is a rational reflection of the problem of generating test data for multiple paths coverage. On this basis, a multipopulation GA is presented to solve the problem in the model. The main idea of this algorithm, very different from traditional multipopulation GAs, is to improve the search efficiency by means of individual sharing among different subpopulations. In addition, we not only prove the efficiency of our method theoretically, but also apply it in various programs under test. The experimental results show that our method has more significant advantages than Ahmed's multiobjective method and random method. The proposed algorithm in this study enriches the theory and technique of GA-based test data generation and provides a new way to improve the efficiency of software testing.

Possible future researches are presented as follows: one is the method to generate test data when the number of target paths is very large; the other one is the establishment of test platform based on our method.

## Figures and Tables

**Table 1:** Branch distances of simple branch conditions [3].

Branch condition	*a* ≥ *b*	*a* > *b*	*a* = *b*	*a* ≠ *b*
True	0	0	0	0
False	*b* − *a*	*b* − *a* + 0.1	|*b* − *a*|	1

**Table 2:** Branch distances of complex branch conditions [3].

Connection	Branch distance
*α* && *β*	*BD*(*α*) + *BD*(*β*)
*α*||*β*	min⁡(*BD*(*α*), *BD*(*β*))

**Table 3:** Description of subject programs.

Program	Loc	Description	Number of targets
Insertion sort	16	Array sorting	4
Bubble sort	18	Array sorting	4
Triangle	20	Return the type of a triangle	4
Binary search	37	Key number searching	7
Gcd	55	Compute greatest common divisor	20
Look	135	Find words in the system dictionary or lines	30
Comm	145	Select or reject lines common	30
Cal	160	Print a calendar for a specified year or month	30
Controller	172	Internal states regulation	30
Tcas	173	Altitude separation	30
Col	275	Filter reverse paper motions	50
Spline	289	Interpolate smooth curve	50
Tot_info	365	Statistics computation	50
Schedule2	374	Priority scheduler	50
Printtok	400	Lexical analyzer	50
Schedule	412	Priority scheduler	50
Replace	564	Pattern replace	100
Barcode	672	Barcode maker	100

**Table 4:** Parameter settings.

Parameter	Value
Population size	300
Selection operator	Roulette wheel
Crossover operator	One-point crossover
Crossover rate	0.9
Mutation operator	One-point mutation
Mutation rate	0.3
Maximum generation	50000
Encoding style	Binary
Variable range	[0, 1023]

**Table 5:** Number of evaluations and success rate of different fitness functions.

Programs	Sharing			No sharing	
	Ave. (s)	S.D.	Sh.R (%)	Ave. (s)	S.D.
Insertion sort	17.62	4.24	34.6	24.74	5.91
**Bubble sort**	**6.38**	**3.72**	**45.6**	**11.03**	**4.93**
Triangle	12.92	5.72	41.3	20.83	7.73
Binary search	39.82	8.18	32.4	48.49	10.93
Gcd	32.74	7.25	36.7	43.83	8.89
Look	16.26	6.32	31.8	22.63	7.83
Comm	30.77	11.86	45.6	50.35	16.37
Cal	39.27	9.06	39.3	55.92	12.83
Controller	46.72	11.72	33.9	63.52	12.89
Tcas	38.62	9.29	32.7	46.83	10.94
Col	75.61	16.72	35.3	103.73	24.65
Spline	50.72	17.75	37.8	69.93	23.89
Tot_info	79.92	20.85	37.9	121.78	26.78
Schedule2	73.88	24.25	31.8	97.62	30.57
Printtok	36.83	15.73	33.7	48.29	19.82
Schedule	45.74	17.99	29.8	65.23	18.74
Replace	141.23	44.27	41.3	176.34	42.87
Barcode	183.53	39.83	39.4	265.72	47.31

**Table 6:** Values of statistic *U*_1_ and *U*_2_ of object programs.

Programs	Values of *U*
Insertion sort	−5.36
Bubble sort	−4.12
Triangle	−4.51
Binary search	−3.48
Gcd	−5.30
Look	−3.47
Comm	−5.31
Cal	−5.81
Controller	−5.28
Tcas	−3.13
Col	−5.17
Spline	−3.54
Tot_info	−6.76
Schedule2	−3.33
Printtok	−2.48
Schedule	−4.11
Replace	−3.12
Barcode	−7.28

**Table 7:** Number of evaluations and success rate of different fitness functions.

Programs	Our method		Gong's method		Ahmed's method		Random method		*U*_1_	*U*_2_	*U*_3_
	Ave. (s)	S.D.	Ave. (s)	S.D.	Ave. (s)	S.D.	Ave. (s)	S.D.			
Insertion sort	19.52	5.07	24.72	6.08	27.93	6.86	42.89	10.82	−3.6	−5.40	−10.71
Bubble sort	5.85	4.29	8.79	4.15	9.85	3.93	12.32	4.17	−2.7	−3.77	−5.92
Triangle	13.58	5.29	17.94	5.12	20.82	7.73	28.73	8.72	−3.24	−4.23	−8.14
Binary search	36.04	10.82	43.24	9.08	54.84	10.93	59.61	13.36	−2.79	−6.70	−7.51
Gcd	34.82	9.93	42.79	9.67	51.37	8.72	72.71	13.49	−3.15	−6.86	−12.39
Look	18.02	7.88	22.94	7.26	25.72	9.79	31.82	10.9	−2.52	−3.36	−5.62
Comm	33.92	12.89	39.74	16.23	50.35	17.48	48.28	15.51	−1.54	−4.14	−3.90
Cal	35.92	10.02	60.93	13.19	60.52	13.07	82.73	15.33	−8.27	−8.18	−14.00
Controller	49.93	12.78	62.19	12.98	71.81	13.83	90.26	15.53	−3.69	−6.36	−10.98
Tcas	35.92	9.95	42.85	9.74	52.89	11.44	77.28	19.53	−2.73	−6.13	−10.34
Col	69.01	15.94	84.23	18.24	112.56	23.78	108.92	26.72	−3.44	−8.33	−7.03
Spline	53.88	17.23	63.99	21.43	78.81	21.84	89.51	20.84	−2.01	−4.91	−7.22
Tot_info	84.27	21.89	114.23	23.14	125.83	29.78	147.34	32.25	−5.15	−6.16	−8.86
Schedule2	78.83	22.51	93.66	26.69	110.73	35.75	135.37	46.31	−2.33	−4.14	−6.01
Printtok	30.72	16.92	35.74	17.83	56.83	20.83	72.75	21.56	−1.12	−5.33	−8.40
Schedule	44.93	15.84	56.84	19.23	70.72	23.82	107.32	21.78	−2.62	−4.94	−12.69
Replace	137.38	40.03	160.98	40.92	188.78	51.78	205.27	48.9	−2.26	−4.30	−5.89
Barcode	192.82	40.17	224.87	49.23	287.67	56.13	316.42	68.73	−2.76	−7.53	−8.50
